# Endometrial regeneration cell-derived exosomes loaded with siSLAMF6 inhibit cardiac allograft rejection through the suppression of desialylation modification

**DOI:** 10.1186/s11658-024-00645-y

**Published:** 2024-10-01

**Authors:** Yini Xu, Shaohua Ren, Hongda Wang, Yafei Qin, Tong Liu, Chenglu Sun, Yiyi Xiao, Bo Shao, Jingyi Zhang, Qiang Chen, Pengyu Zhao, Guangmei Yang, Xu Liu, Hao Wang

**Affiliations:** 1 Department of General Surgery, Tianjin Medical University General Hospital, 154 Anshan Road, Heping District, Tianjin, 300052 China; 2 Tianjin General Surgery Institute, 154 Anshan Road, Heping District, Tianjin, 300052, China; 3 Tianjin Key Laboratory of Precise Vascular Reconstruction and Organ Function Repair, 154 Anshan Road, Heping District, Tianjin, 300052 China; 4 Department of Vascular Surgery, Henan Provincial People’s Hospital, The Affiliated People’s Hospital of Zhengzhou University, Zhengzhou, 450003 Henan China

**Keywords:** Signaling lymphocyte activation molecule family 6 (SLAMF6), Cardiac allograft rejection, Endometrial regeneration cell-derived exosome, Desialylation, Modification

## Abstract

**Backgrounds:**

Acute transplant rejection is a major component of poor prognoses for organ transplantation. Owing to the multiple complex mechanisms involved, new treatments are still under exploration. Endometrial regenerative cells (ERCs) have been widely used in various refractory immune-related diseases, but the role of ERC-derived exosomes (ERC-Exos) in alleviating transplant rejection has not been extensively studied. Signaling lymphocyte activation molecule family 6 (SLAMF6) plays an important role in regulating immune responses. In this study, we explored the main mechanism by which ERC-Exos loaded with siSLAMF6 can alleviate allogeneic transplant rejection.

**Methods:**

C57BL/6 mouse recipients of BALB/c mouse kidney transplants were randomly divided into four groups and treated with exosomes. The graft pathology was evaluated by H&E staining. Splenic and transplanted heart immune cell populations were analyzed by flow cytometry. Recipient serum cytokine profiles were determined by enzyme-linked immunosorbent assay (ELISA). The proliferation and differentiation capacity of CD4^+^ T cell populations were evaluated in vitro. The α-2,6-sialylation levels in the CD4^+^ T cells were determined by SNA blotting.

**Results:**

In vivo, mice treated with ERC-siSLAMF6 Exo achieved significantly prolonged allograft survival. The serum cytokine profiles of the recipients were significantly altered in the ERC-siSLAMF6 Exo-treated recipients. In vitro, we found that ERC-siSLAMF6-Exo considerably downregulated α-2,6-sialyltransferase (ST6GAL1) expression in CD4^+^ T cells, and significantly reduced α-2,6-sialylation levels. Through desialylation, ERC-siSLAMF6 Exo therapy significantly decreased CD4^+^ T cell proliferation and inhibited CD4^+^ T cell differentiation into Th1 and Th17 cells while promoting regulatory T cell (Treg) differentiation*.*

**Conclusions:**

Our study indicated that ERC-Exos loaded with siSLAMF6 reduce the amount of sialic acid connected to α-2,6 at the end of the N-glycan chain on the CD4^+^ T cell surface, increase the number of therapeutic exosomes endocytosed into CD4^+^ T cells, and inhibit the activation of T cell receptor signaling pathways, which prolongs allograft survival. This study confirms the feasibility of using ERC-Exos as natural carriers combined with gene therapy, which could be used as a potential therapeutic strategy to alleviate allograft rejection.

**Supplementary Information:**

The online version contains supplementary material available at 10.1186/s11658-024-00645-y.

## Background

Organ transplantation is the most effective treatment for end-stage organ failure [1]. Acute transplant rejection is one of the most prevalent problems following transplantation, and it has a substantial effect on the function of transplanted organs as well as patient survival time [2]. Given that long-term and intensive use of conventional immunosuppressants has significant unfavorable effects [3–5], novel alternative therapeutic strategies must be explored. Signaling lymphocyte activation molecule family 6 (SLAMF6), a member of the immunoglobulin superfamily [6], is expressed on the surface of various immune cells [7]. It plays a critical role in regulating both innate and adaptive immune responses, including T lymphocyte proliferation, natural killer cell activation, and CD8^+^ T cell-mediated cytotoxicity [8, 9]. Whole-genome RNA sequencing has identified SLAMF6 as a potential molecular marker for detecting acute rejection in allogeneic renal transplantation [10]. Additionally, the adoptive transfer of SLAMF6^−/−^ CD4^+^ T cells into bm12 mice has been shown to significantly attenuate the severity of autoimmune reactions [11]. These findings suggest that SLAMF6 plays a critical role in immune regulation and may serve as an important therapeutic target.

Exosomes derived from endometrial regenerative cells (ERC-Exosomes) have emerged as a safer therapeutic strategy for treating organ transplantation. Exosomes cause little immunological rejection and can carry biological genetic information across the blood–brain barrier [12]. Therefore, exosomes are considered ideal small interfering RNA (siRNA) carriers. Integrating exosomes with gene therapy has emerged as a primary strategy to increase the therapeutic efficacy of stem cell-derived exosomes [13].

Glycosylation is a key component of protein posttranslational modification, and aberrant glycosylation can be pro- or anti-inflammatory. Many studies have shown that aberrant glycosylation plays an important role in the development of autoimmune diseases [14]. Sialylation is a type of glycosylation, and each type of sialylation is catalyzed by its corresponding sialyltransferases (STs), which transfer sialic acid from the donor to the acceptor glycan. Research has shown that sialylation impacts immunological responses and the development of intestinal inflammation [15]. Previous research has employed mass spectrometry to examine the sugar chain structure of exosomes derived from T lymphocytes and tumor cells, and it has shown that these exosomes are considerably enriched in mannose and α-2,6 sialic acid-type sugar chains [16]. However, the role of sialylation in acute transplant rejection requires further investigation.

In this study, we showed that ERC-Exos loaded with siSLAMF6 play a role in desialylation modification by reducing the sialic acid at the end of the N-sugar chain connected to the cell surface by α-2,6 bonds, thereby increasing the number of exosomes endocytosed into CD4^+^ T cells. They prevent the activation of the TCR signaling pathway and the binding of RORγT to the interleukin (IL)-17A promoter region, thereby inhibiting the proliferation of CD4^+^ T cells and their differentiation into Th1 and Th17 cells and promoting the generation of regulatory T cells (Tregs), thereby effectively inhibiting the occurrence of acute transplant rejection.

## Materials and methods

### ERC extraction and siRNA transfection

ERC separation was performed according to a previously described protocol [17, 18]. In brief, ERCs were isolated from the menstrual blood of 20–40-year-old healthy female volunteers on the second day of their menstrual cycles. The menstrual blood sample collection procedure was approved by the Medical Ethics Committee of Tianjin Medical University General Hospital (IRB2023-WZ-173, Tianjin, China). To obtain mononuclear cell layer cells, the menstrual blood was washed with culture media, filtered through a 200 μm mesh filter, and centrifuged with a Ficoll solution gradient. The cells were harvested and resuspended in Dulbecco’s modified Eagle's medium/nutrient mixture F-12 (DMEM–F12) (HyClone, USA) supplemented with 15% fetal bovine serum (FBS) (Corning, New Zealand). The cells were incubated at 37 °C, in a 5% CO_2_ incubator. When the cells reached 80% confluence, they were digested and passaged with trypsin solution (Solarbio, China), and the culture medium was changed every 2 or 3 days.

Before the siRNA transfection of ERCs, the cells were equally distributed within a six-well plate. When the cell density reached 60–80%, the cells were transfected with the GP-transfect-mate transfection reagent according to the manufacturer’s recommendations. The culture medium was changed after 6 h of culture. The target sequences for si-NC were as follows: sense sequence (5′–3′): UGACCUCAACUACAUGGUUTT; antisense sequence (5′–3′): AACCAUGUAGUUGAGGUCATT. The target sequences for si-SLAMF6 were as follows: sense sequence (5′–3′): GGCAACUGCCCUUGACAAUTT; antisense sequence (5′–3′): AUUGUCAAGGGCAGUUGCCTT.

### Exosome extraction

Gradient differential centrifugation was used to isolate exosomes from the cell culture medium. Before the cell culture medium was collected, it was changed to serum-free medium for 72 h. The samples were centrifuged at 300*g*, 3000*g*, and 10,000*g* after collection to remove any cell debris and other larger-sized extracellular vesicles. Lastly, the culture medium was centrifuged for 70 min in an ultracentrifuge at 100,000*g*. The exosomes were resuspended in 1× phosphate-buffered saline (PBS) and stored at 4 °C for 1 week. The exosome proteins were resuspended in cell lysis buffer for extraction.

### Flow cytometry

Flow cytometry was employed to identify ERCs and quantify the CD4^+^ T cell populations, both in vitro and in vivo. To obtain splenocytes, the recipient spleens were collected, smashed, and lysed to obtain red blood cells. Percoll density gradient centrifugation was used to isolate cardiac mononuclear cells from heart transplants. Before surface staining, the cells were stained with Zombie NIR (BioLegend, USA) to distinguish dead cells from live cells for ERC identification. Following surface labeling, the cell membrane was perforated with a fixation/permeabilization kit (Thermo Fisher Scientific) for intracellular staining. When evaluating Th1 and Th17 cells, cell-stimulating agents must be used 5 h in advance to stimulate early cytokine synthesis and block cytokine secretion. The stained cell suspension was kept in 1% paraformaldehyde for additional use. All the fluorescent antibodies used in this study were obtained from eBioscience. The panel comprised the following anti-human antibodies: CD45-PE, HLA-DR-PE, CD73-FITC, CD44-APC, CD79-PE, and CD90-PE, as well as the following anti-mouse antibodies: CD4-FITC, IFNγ-PE, IL-17A-Percp-Cyanine 5.5, CD25-PE, and Foxp3-APC.

### Cell immunofluorescence

First, CD4^+^ T cells were isolated by magnetic bead sorting. Exosomes were then labeled with a PKH26 red fluorescence staining kit (Sigma, USA). The labeled exosomes were co-incubated with CD4^+^ T cells according to the manufacturer’s instructions. Owing to the features of the T cell suspension, the cells were evenly dispersed on a polylysine-coated glass slide after incubation and dried in a 37 °C cell culture incubator. Images were taken with a confocal microscope after mounting media containing DAPI (Abcam, UK) was applied.

### Western and lectin blot assays

Total cellular protein was extracted with lysis buffer, protease, and phosphatase inhibitors, and the protein concentration was determined with a Nanodrop 1000 ultramicro spectrophotometer. After being treated with β-mercaptoethanol, the proteins were separated using sodium dodecyl-sulfate–polyacrylamide gel electrophoresis (SDS–PAGE) gel electrophoresis. The gel was transferred to a polyvinylidene difluoride (PVDF) membrane after performing protein electrophoresis at 300 mA constant current, blocked for 1 h at room temperature with 5% skim milk, and then incubated overnight at 4 °C with diluted primary antibody. The next day, the PVDF membrane was stripped with tris-buffered saline with tween-20 (TBST), the appropriate secondary antibody was added, and streptavidin was used as the secondary antibody for the lectin blot. After 1 h of incubation at room temperature, bioluminescence was detected using a chemiluminescence reagent.

### Molecular docking

The Protein Data Bank (PDB) (https://www.rcsb.org) was used to find the protein IDs corresponding to SLAMF6 and SH2D1A, the two protein IDs in ZDOCK SERVER (https://zdock.wenglab.org) were entered, and the docking conformations of the two proteins were obtained. The interaction force of SLAMF6 and SH2D1A binding was predicted and visualized using PDBePISA (https://www.ebi.ac.uk/msd-srv/prot_int/).

### Coimmunoprecipitation (co-IP)

Total cellular protein was extracted with lysis buffer containing protease and phosphatase inhibitors. For co-IP, cellular proteins were coated with antibodies overnight at 4 °C before being incubated with protein A/G agarose beads (Sigma, USA) for 2 h the next day. After with the beads were washed with lysis buffer, the protein levels were determined using a western blot analysis. The remaining steps were the same as those for the western blot analysis.

### Chromatin immunoprecipitation

Chromatin immunoprecipitation was performed according to the manufacturer’s instructions (Sigma, USA). In brief, protein–DNA complexes were fixed in living cells, randomly fractured into small fragments of 200–1000 bp by ultrasonic disruption, and then precipitated with specific antibodies. The target protein-bound DNA fragment was subsequently isolated, and the purified products were evaluated using agarose gel electrophoresis.

### Luciferase reporter assay

The promoter sequences for IL-17A and IL-22 were retrieved from the the NCBI database (https://www.ncbi.nlm.nih.gov/) and subsequently cloned and inserted into the pGL3-basic plasmid. To predict the binding sites of RORγt on the IL-17A and IL-22 promoters, the JASPAR database (https://jaspar.genereg.net/) was used. HEK-293T cells were seeded into 96-well plates and transfected once they reached 80% confluence. The IL-17A and IL-22 promoter constructs, along with the control Renilla luciferase reporter plasmid, were introduced into the cells using the Lipofectamine 3000 reagent according to the manufacturer’s protocol. The luciferase activity was then quantified using a dual luciferase reporter assay system (Promega).

### Enzyme-linked immunosorbent assay (ELISA)

The culture supernatants of ERCs and ERCs transfected with siSLAMF6 were collected, and the secretions of IL-4, IFNγ, and IL-17A were measured using an ELISA kit (DAKEWE, China) according to the manufacturer’s instructions.

### Heterotopic cardiac transplantation

An abdominal heterotopic heart transplantation was performed using surgical procedures as we described previously [19]. BALB/c and C57BL/6 mice of similar ages and weights were selected as donors and recipients, respectively. All the animal experiments were approved by the Animal Experimentation Ethics Committee of Tianjin Medical University General Hospital. All of the experiments were performed in accordance with the Basel Declaration. Prior to modeling, all the mice were housed adaptively for at least 1 week. The breeding environment was maintained under pathogen-free conditions, with a stable temperature and humidity, a 12 h light–dark cycle, and a balanced nutritional diet available ad libitum, along with access to clean, sterile drinking water. To extract the donor hearts, the BALB/c mice were first anesthetized, followed by sternotomy to expose and separate the inferior vena cava and abdominal aorta, which were then heparinized. The superior vena cava and pulmonary vein were ligated and severed, after which the ascending aorta and pulmonary artery were transected, allowing for the removal of the donor heart. The heart was then temporarily stored in organ preservation fluid. For the transplantation procedure, the ascending aorta and pulmonary artery of the donor heart were end-to-side anastomosed to the abdominal aorta and inferior vena cava in the recipient C57BL/6 mice, respectively. Upon the release of the vascular clamps, the donor heart typically resumed beating within 1–2 min, becoming uniformly bright red. Posttransplantation, the recipient mice were kept in a warm environment and housed individually. The pulsation of the transplanted hearts was monitored daily by abdominal palpation.

The mouse recipients were randomly divided into four groups (*n* = 6 for each group), namely the untreated group, the ERC-siNC Exo group, the ERC-Exo group, and the ERC-siSLAMF6 Exo group. One day before surgery, 100 µg of exosomes were injected into the tail veins of the mice in the treatment group. On the third and fifth days postsurgery, 100 µg of exosomes was also injected through the tail vein. For the mice used in the survival analysis, either complete cardiac arrest or postoperative day 100 (POD100) was set as the endpoint. The remaining mice were sacrificed on postoperative day 7 (POD7) for subsequent experimental procedures.

### Isolation of mononuclear cells from allografts

Allografts were harvested on the seventh day after surgery and placed in heparinized saline. The epicardial adipose tissue was removed, the allograft was minced, and residual blood was repeatedly flushed with heparinized saline. Minced tissues were treated with 2 mg/mL type II collagenase (Solarbio, China), 100 μg/mL DNase I (Solarbio, China), and 0.1% trypsin (Solarbio, China). For digestion, 5 mL of RPMI 1640 medium (HyClone, USA) was added, and the mixture was incubated in a shaker at 37 °C for 90 min. Next, an equal volume of serum-containing medium was added to terminate the digestion. The cells were harvested after being filtered through a 70 μm cell strainer and centrifuged at 300*g* for 5 min at 4 °C. The cell pellet was resuspended in different concentrations of Percoll. After centrifugation, the mononuclear cells in the middle white layer were collected for subsequent flow cytometric analysis.

### Hematoxylin–eosin (H&E) staining

Allograft heart tissue was collected and stored in 10% formalin. The samples were fixed in paraffin 2 days later and cut into 5 μm slices. After the slices were dewaxed and rehydrated, the nuclei were stained with hematoxylin, and the cytoplasm was stained with eosin. The slices were mounted after dehydration. Images were captured using an optical microscope after natural air drying. The statistics were based on the H&E scores. The pathological grading of transplant rejection follows the 2004 revision of the International Society of Heart and Lung Transplantation (ISHLT) diagnostic criteria for rejection [20], i.e., classifying rejection into four grades: Grade 0 indicates no rejection, with normal myocardial cells; Grade 1 represents mild rejection, characterized by interstitial and/or perivascular inflammatory infiltration; Grade 2 denotes moderate rejection, with multifocal diffuse inflammatory infiltration accompanied by myocyte damage; and Grade 3 signifies severe rejection, marked by diffuse infiltration with multifocal myocyte damage ± edema, ± hemorrhage ± vasculitis.

### Bioinformatics analysis

Gene expression profiles were queried in the Gene Expression Omnibus (GEO) database (https://www.ncbi.nlm.nih.gov/geo/) using the keyword “acute transplant rejection.” The dataset GSE46474 was identified, comprising 20 patients who experienced acute transplant rejection within 30 days posttransplantation (AR patients) and 20 controls who also received a transplant but did not experience acute rejection for at least 6 months posttransplant (NR patients). Peripheral blood samples from these patients were analyzed using a whole-genome microarray. Notably, there were no significant differences in age, sex, race, or donor type between the two patient groups.

The limma package of R software was used to study the differential expression of mRNAs for each group [21]. Adjusted *P* < 0.05 and log_2_FC > 0.5 or log_2_FC < −0.5 was defined as a differential gene. The data from patients in the NR group were used as controls. Genes with normalized log_2_ fold change (FC) values greater than 0.5 were classified as upregulated, whereas those with log_2_FC values of less than −0.5 were classified as downregulated. Heat maps and volcano plots were generated using the pheatmap package in R to visualize these differentially expressed genes. The Kyoto Encyclopedia of Genes and Genomes (KEGG) pathways enriched in differentially expressed genes were visualized using the online bioinformatics annotation tool Hiplot pro (https://hiplot.com.cn/). Pathways were considered significantly enriched if they met the cutoff criteria of a *P* value < 0.05 and a false discover rate (FDR) < 0.05. Immune cell infiltration analysis is important for predicting disease course and treatment response. CIBERSORT can be used to estimate the number of immune cells within a sample [22]. We employed the CIBERSORT algorithm in R to assess the relative abundance of 22 distinct immune cell types across the two patient groups. The results were visualized using box plots, providing a comparative overview of the immune cell composition between the groups.

### Statistical analysis

In this study, each experiment was repeated three times independently. The mean and standard deviation (SD) were used to display the results. GraphPad Prism 9.0 software was used for statistical analysis. One-way analysis of variance (ANOVA) was used to evaluate data within groups, and a *t*-test was used to evaluate data between two groups. Differences were considered statistically significant at *P* < 0.05.

## Results

### Expression characteristics of acute transplant rejection analyzed using the GEO database

**Fig. 1:**
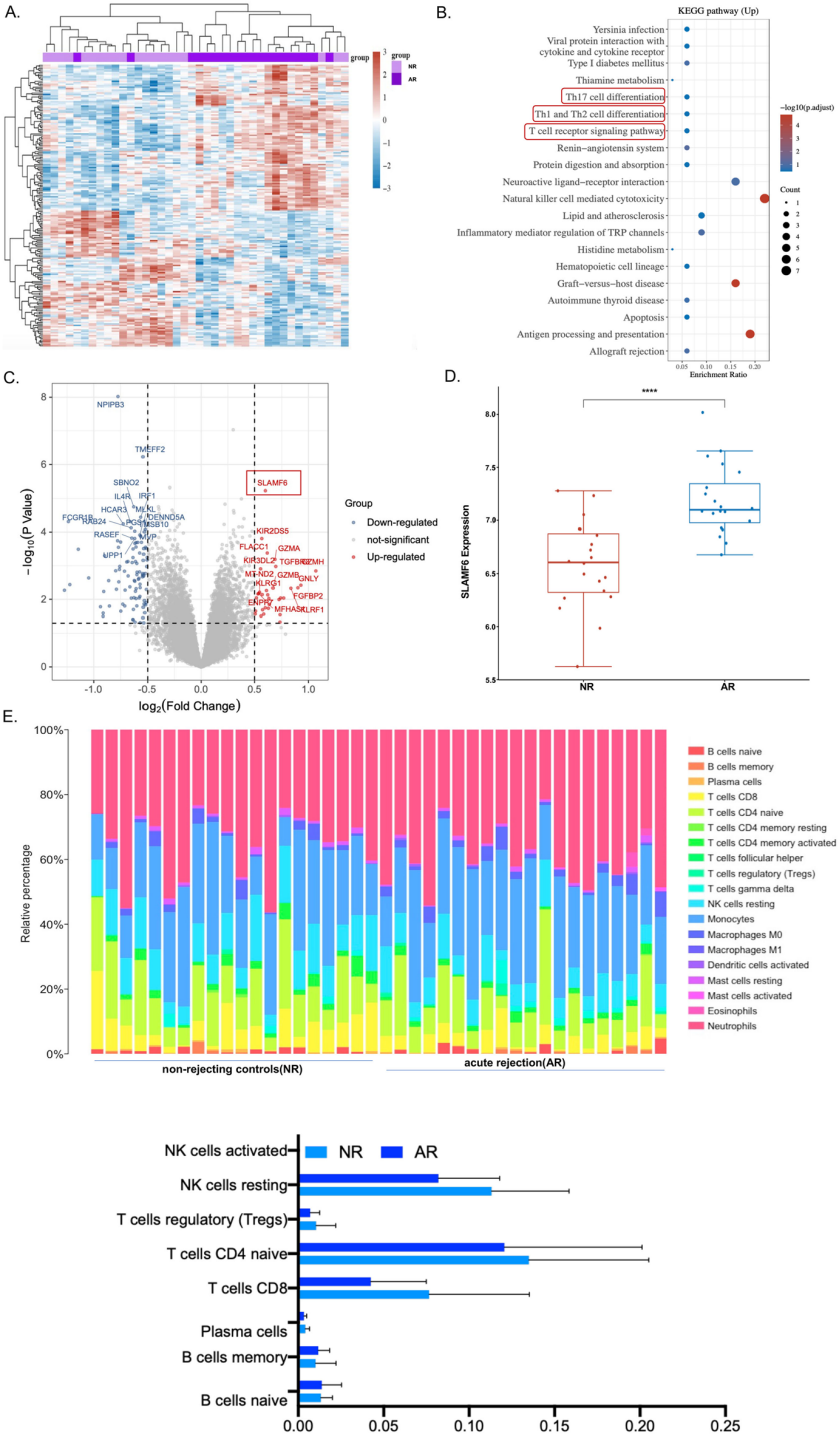
The GEO database was used to investigate the expression characteristics of acute transplant rejection. **A** Differential gene expression heat map, in which different colors represent trends in gene expression for different tissues. This graph displays the top 50 upregulated genes and the top 50 downregulated genes. Each column on the horizontal axis represents a sample, light purple represents patients in the NR group, and dark purple represents patients in the AR group. The vertical axis on the left cluster different genes. The color scale changes on the right represent the level of differential gene expression; red indicates upregulation, and blue indicates downregulation. **B** KEGG pathway analysis revealed the top 20 pathways associated with the DEGs. The *x*-axis denotes the degree of pathway enrichment, whereas the *y*-axis represents the functional pathways. The color of the bubbles indicates the significance of the enrichment, and the size of the bubbles corresponds to the number of genes significantly enriched in the respective pathways. **C** A volcano plot was constructed using the fold change values and *P* values. Red dots indicate upregulated genes and blue dots indicate downregulated genes. **D** Distribution of SLAMF6 expression in different groups. The abscissa represents several groups of samples, while the ordinate indicates the gene expression distribution; different colors denote various groups (acute rejection versus nonrejecting controls,* P* < 0.0001). The Wilcoxon test was used to evaluate the significant differences between the two groups. **E** The proportions of infiltrating immune cells in the acute rejection and nonrejection groups were compared on the basis of CIBERSORT. The fraction of cells is shown on the ordinate, whereas each sample in different groups is shown on the abscissa. The statistics for visualization are provided below

To explore the potential factors determining rejection after transplantation, we first screened and analyzed the Gene Expression Omnibus (GEO) public database, acquiring GSE46474 expression data in acute rejection (*n* = 20) and nonrejecting controls (*n* = 20). Moreover, we analyzed the patient information provided by the public database and confirmed that there were no significant differences in age, sex, race, or donor type (Supplementary Table 1). After the data were normalized, the enrichment statistics of the differentially expressed genes were calculated (Fig. 1A). Light purple indicates the normal control samples (NR), while dark purple denotes the disease samples (AR). Red represents upregulated differentially expressed genes (DEGs), whereas blue signifies downregulated DEGs. The DEGs upregulated in patients with transplant rejection compared with patients without transplant rejection were analyzed using KEGG, and the upregulated genes were largely enriched in the 20 pathways shown in Fig. 1B. The results of the KEGG pathway analysis are consistent with those of earlier studies, namely, that Th17 cell differentiation, Th1 and Th2 cell differentiation, and T cell receptor signaling pathways are associated with acute transplant rejection [23–25]. The distribution of differential gene expression was subsequently analyzed, with red indicating upregulated DEGs and blue representing downregulated DEGs (Fig. 1C). SLAMF6 expression was considerably increased in patients with graft rejection (Fig. 1D). The CIBERSORT technique was used to analyze immune infiltration, and the proportion of immune cells in each sample was determined (Fig. 1E). The findings revealed that NK cells, neutrophils, and monocytes were the most critical immune cells. Compared with nonrejected controls, NK cells, CD4^+^ T cells, and CD8^+^ T cells were the most abundant immune cells in acute rejection samples. Acute transplant rejection is an acute immunological response mediated by T cells, most notably CD4^+^ T lymphocytes [26]. Although B cell-mediated immune rejection plays an important role after transplantation, SH2D1A, the intracellular adapter of SLAMF6, is expressed only on T cells (not B cells). Given this characteristic, our research focused on the relationship between SLAMF6 and CD4^+^ T cell differentiation in transplant rejection.

### Establishment and characterization of ERC-derived exosomes loaded with siSLAMF6

**Fig. 2:**
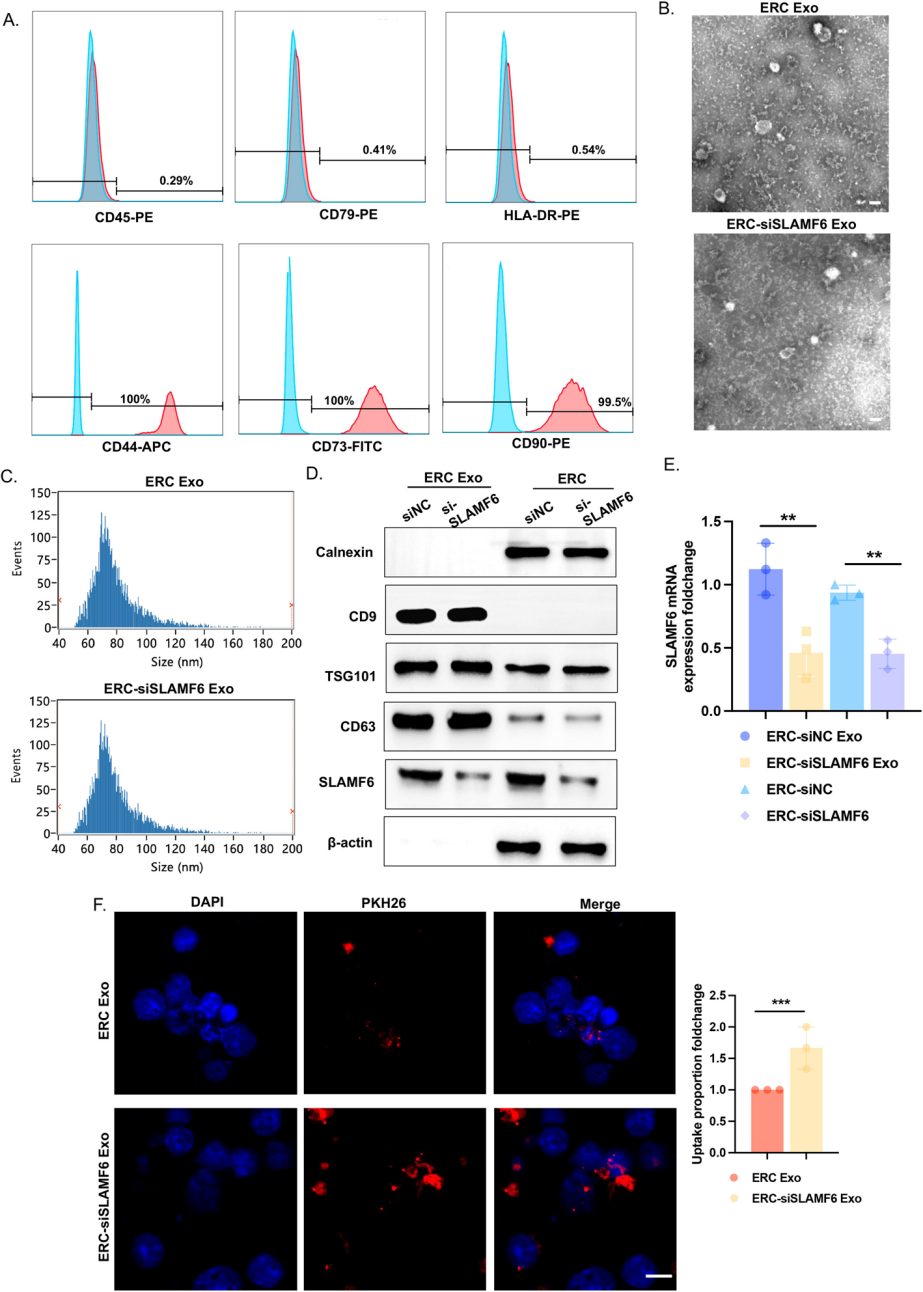
Establishment and characterization of ERC-derived exosomes loaded with siSLAMF6. **A** Flow cytometry was used to identify the expression of the positive markers CD44, CD73, and CD90 and the negative markers CD45, CD79, and HLA-DR on the surface of the ERC cells. **B** Transmission electron microscopy (TEM) confirmed that the morphology and size of the ERC-Exos were consistent with those of ERC-siSLAMF6 Exos. Scale bars = 100 nm. **C** Nanoparticle tracking analysis (NTA) was used to determine the particle size distributions of ERC-Exo and ERC-siSLAMF6 Exo. **D** Western blot analysis was performed using SLAMF6 and the exosome-positive markers CD9, TSG101, and CD63 and the negative marker calnexin to verify the purification of the exosomes and the knockdown efficiency of siSLAMF6. **E** mRNA levels of SLAMF6 in ERC, ERC-siSLAMF6, ERC-Exo, and ERC-siSLAMF6 Exo (**P < 0.01). **F** Confocal microscopy was used to detect the uptake of PKH26-labeled Exos by T cells after they were incubated with T cells. The left side shows representative images, and the right side shows the changes in the proportion of exosome uptake per 20 cells. Scale bars = 100 μm (***P < 0.001)

When ERCs grew to the third generation, surface markers were identified, and CD44, CD73, and CD90, but not CD45, CD79, or HLA-DR, were expressed on the surface of the ERCs, which demonstrated that the ERCs expressed stem cell surface markers (Fig. 2A). Exosomes derived from stem cells have been shown to treat transplant rejection [27], while exosomes, as natural nanocarriers, can transport siRNA for therapeutic effects. We transfected ERCs with siRNA and isolated ERC-derived exosomes (ERC-Exo) and ERC-siSLAMF6-derived exosomes (ERC-siSLAMF6 Exo) by gradient differential centrifugation. Transmission electron microscopy (TEM) revealed that the exosomes in both groups had small round or oval vesicles, suggesting that transfection did not affect the exosome morphology. Nanoparticle tracking analysis (NTA) revealed that the diameters of the two types of exosomes were substantially different. The average diameter of the exosomes in the control group was 78.62 nm, whereas the average for the treatment group was 78.66 nm. These findings confirmed that transfection had little effect on the exosome size (Fig. 2B, C).

In accordance with the requirements of the International Society for Extracellular Vesicles, exosome identification includes at least three positive protein markers and one negative protein marker, and the positive protein markers require one transmembrane protein and one cytosolic protein. We used western blotting to reveal that both ERC-Exos and engineered ERC-Exos highly expressed CD9, TSG101, and CD63 and expressed low levels of calnexin. These findings indicate that engineering the ERC does not affect the expression of exosome markers. We also found that the expression level of SLAMF6 was high in ERC and ERC-Exos, which explains why our groups previous use of ERC alone to treat transplant rejection was not efficient, perhaps because the ERC itself contains genes that promote transplant rejection, including SLAMF6 (Fig. 2D). Moreover, we confirmed that the transfection of siSLAMF6 also significantly decreased the SLAMF6 mRNA levels in ERC and ERC-Exos (Fig. 2E).

PKH26 is a lipophilic dye that may attach to the lipid area of the cell membrane and emit red fluorescence when stably bound to it. To validate the affinity of differently treated exosomes for recipient cells, we labeled exosomes with PKH26 and cocultured differently treated exosomes with T cells for 3 h. The findings demonstrated that after transfection with siSLAMF6, recipient cells were able to take up more exosomes (Fig. 2F). These findings demonstrate that siRNA transfection not only has no effect on exosome quality or quantity but also allows more exosomes to reach recipient cells while retaining therapeutic benefits.

### ERC-Exo loaded with siSLAMF6 suppressed the proliferation of T cells and the activation of the T cell receptor (TCR) signaling pathway

**Fig. 3:**
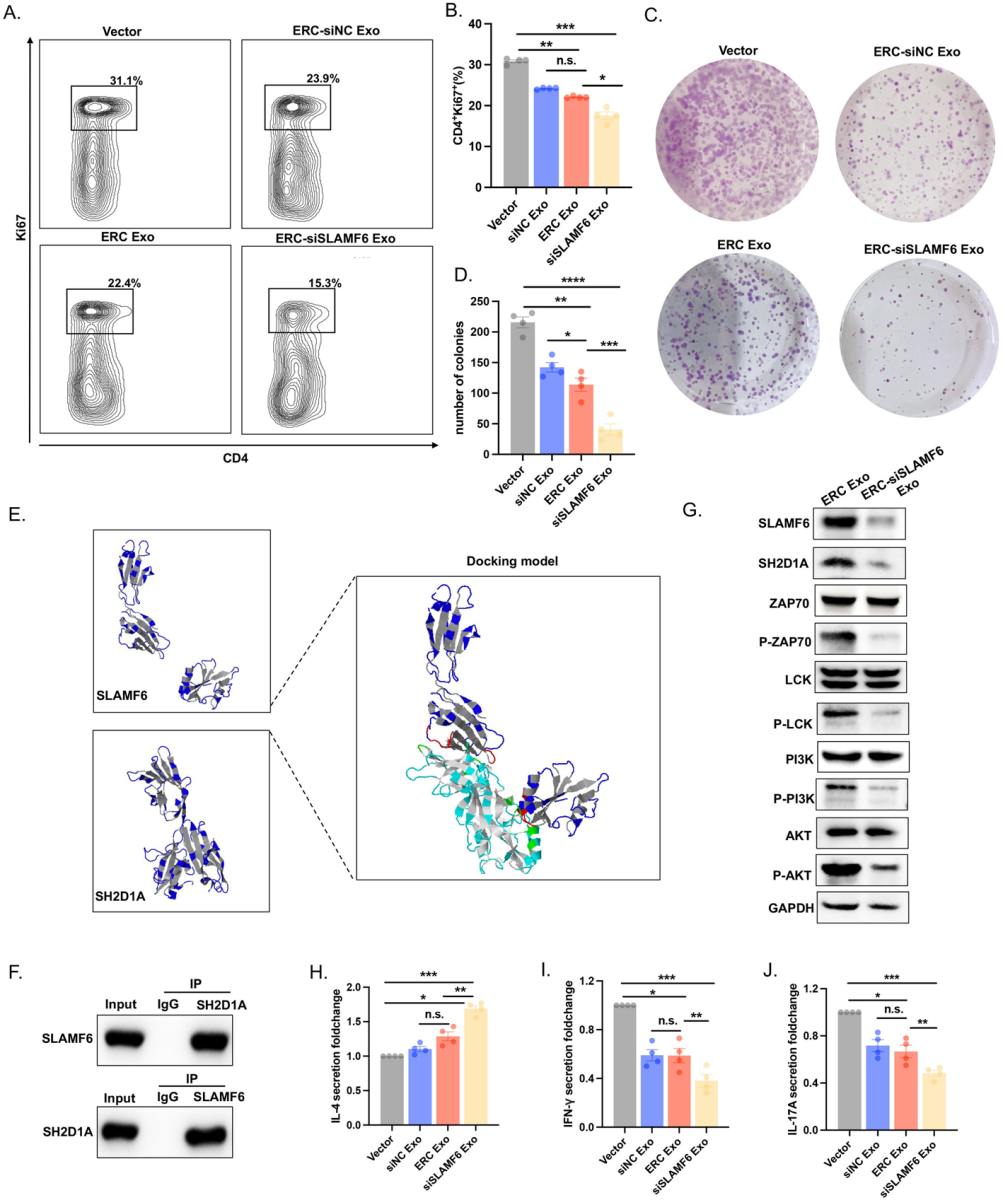
ERC-Exo loaded with siSLAMF6 suppressed the proliferation of T cells and the activation of the T cell receptor signaling pathway. **A** Flow cytometry was used to calculate the percentage of CD4^+^Ki67^+^ T cells after 48 h of coculture with exosomes from different groups. The vector represents CD4^+^ T cells that did not receive any treatment. **B** The histogram represents the change in the percentage of CD4^+^Ki67^+^ T cells (ERC-siSLAMF6 Exo group versus vector group, *P* < 0.001; ERC-Exo group versus ERC-siNC Exo group, *P* > 0.05; ERC-siSLAMF6 Exo group versus ERC-Exo group, *P* < 0.05; and ERC-Exo group versus vector group, *P* < 0.01). **C** A colony formation assay was used to investigate the proliferation ability of CD4^+^ T cells cocultured with different groups of exosomes. **D** The histogram presents the number of CD4^+^ T cells (ERC-siSLAMF6 Exo group versus vector group, *P* < 0.0001; ERC-Exo group versus ERC-siNC Exo group, *P* < 0.05; ERC-siSLAMF6 Exo group versus ERC-Exo group, *P* < 0.001; ERC-Exo group versus vector group, *P* < 0.01). **E** The molecular docking model predicted the binding region of SLAMF6 and SH2D1A. **F** Co-IP assays revealed SLAMF6 and SH2D1A interactions in T cells. **G** Western blotting was used to detect the expression of SH2D1A, ZAP70, P-ZAP70, LCK, P-LCK, PI3K, P-PI3K, AKT, and P-AKT in the T cell receptor signaling pathway (TCR pathway) in CD4^+^ T cells cocultured with ERC-Exos or ERC-siSLAMF6 Exos for 48 h. GAPDH was used as the loading control. **H**–**J** ELISA was used to evaluate the levels of IL-4, IFNγ, and IL-17A secreted by the cell culture supernatant after 48 h of coculture with different groups of exosomes. n.s., *P* > 0.05, *P < 0.05, **P < 0.01 and ***P < 0.001

Our previous study revealed that transplant rejection is strongly associated with abnormal activation of CD4^+^ T cells [17, 28, 29]. We first collected CD4^+^ T cells from mouse splenocytes using magnetic bead sorting to explore the role of SLAMF6 in CD4^+^ T cell activation. CD4^+^ T cells were cocultured with or without different ERC-derived exosome treatments. Cocultivation with ERC-siNC Exos and ERC-Exos reduced the proportion of Ki67-producing cells compared with that in the vector group, whereas cocultivation with ERC-siSLAMF6 Exos significantly reduced the proportion of Ki67-producing cells (Fig. 3A, B). Moreover, a colony formation assay was performed on cells treated similarly to those shown in Fig. 3B. Compared with no treatment, coculture with ERC-siNC Exo and ERC-Exo reduced CD4^+^ T cell proliferation, whereas coculture with ERC-siSLAMF6 Exo minimized CD4^+^ T cell proliferation (Fig. 3C, D).

As confirmed by previous studies, the participation of SH2D1A is required for SLAMF6 function in T cells. SLAMF6's extracellular domain functions as its autoligand, whereas its intracellular adaptor SH2D1A regulates the activation of T-cell signaling pathways by binding [30, 31]. We used molecular docking to predict whether SLAMF6 and SH2D1A would interact structurally. The results revealed that SLAMF6 can form hydrogen bond interactions with SER90, GLY4009, and other residues of SH2D1A through residues such as ARG86 and GLN88, thus achieving a stable combination effect (Fig. 3E). Coimmunoprecipitation (co-IP) was then used to identify the interaction between SLAMF6 and SH2D1A in the CD4^+^ T cells, and the results were consistent with our predictions (Fig. 3F). As shown in Fig. 1, there was activation of the TCR signaling pathway and high expression of SLAMF6 during transplant rejection, but the relationship between SLAMF6 and the TCR pathway has not yet been verified. Therefore, we collected ERC-Exos and ERC-siSLAMF6 Exos, cocultured them with CD4^+^ T cells, extracted cell proteins, and verified the gene activation in the TCR pathway. Compared with ERC-Exos, ERC-Exos loaded with siSLAMF6 considerably inhibited TCR pathway activation (Fig. 3G).

Additionally, we collected culture supernatants from CD4^+^ T cells that were cocultured with various exosomes. The secretion of the anti-inflammatory factor IL-4 was considerably increased in the ERC-siSLAMF6 group compared with the other three groups (Fig. 3H), whereas the secretion of the proinflammatory factors IFNγ and IL-17A (Fig. 3I, J) was significantly reduced. These results suggest that ERC-Exos loaded with siSLAMF6 could be used as a potential therapeutic strategy for immune regulation. Owing to the limitations of siRNAs in clinical applications, exosomes have been used as delivery vehicles for siSLAMF6, which can prevent allograft rejection by suppressing T-cell proliferation and TCR pathway activation.

### ERC-Exo loaded with siSLAMF6 regulated T-cell differentiation in vitro

**Fig. 4:**
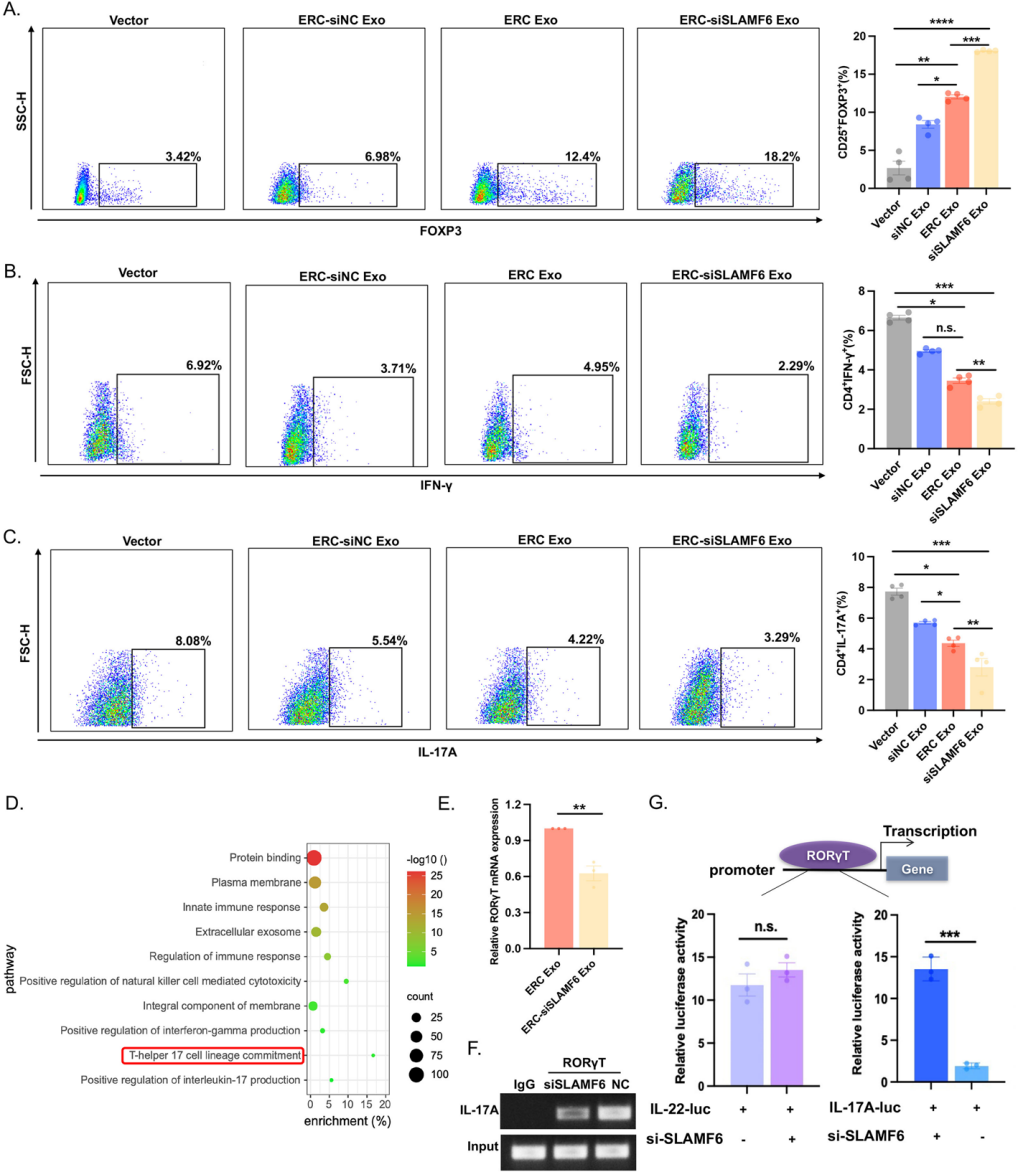
ERC-Exo loaded with siSLAMF6 regulated T cell differentiation in vitro. **A**–**C** Flow cytometry was used to determine the proportions of Treg (CD4^+^CD25^+^FOXP3^+^) cells, Th1 (CD4^+^IFNγ^+^) cells, and Th17 (CD4^+^IL-17A^+^) cells after the CD4^+^ T cells were treated with exosomes. The left panel shows representative images, while the right panel shows a histogram of each groups proportion statistics. n.s., *P* > 0.05, *P < 0.05, **P < 0.01, ***P < 0.001, and ****P < 0.0001. **D** The enriched KEGG signaling pathways were selected to identify the top ten pathways in which SLAMF6 plays a major biological role. **E** RT–PCR analysis was used to determine the influence of SLAMF6 on the expression of RORγT mRNA (ERC-siSLAMF6 Exo group versus ERC-Exo group, *P* < 0.01). **F** Chromatin immunoprecipitation assays revealed that SLAMF6 knockdown impaired RORγT binding to the promoter of IL-17A. **G** The effect of SLAMF6 RORγT on IL-17A transcriptional activity was assessed using a luciferase reporter assay. The influence of the RORγT system on IL-22 transcriptional activity was employed as a positive control. n.s., *P* > 0.05, and ***P < 0.001

CD4^+^ T cells were cocultured with ERC-siNC Exos, ERC-Exos, or ERC-siSLAMF6 Exos to verify that ERC-Exos loaded with siSLAMF6 play an immunomodulatory role in vitro. We found that the proportion of T cells that differentiated into Tregs was significantly greater in the ERC-Exo loaded with siSLAMF6 group than in the other three groups (Fig. 4A). We also found that ERC-siNC and ERC-Exo could both inhibit T-cell development into Th1 cells and that using ERC-siSLAMF6 Exo effectively lowered the proportion of Th1 cells (Fig. 4B). A similar finding was also observed in Th17 cells (Fig. 4C). To confirm that the influence of ERC-siSLAMF6 Exos on CD4^+^ T cell differentiation is attributed primarily to the siSLAMF6 component, we transfected CD4^+^ T cells with siSLAMF6 alone. Our results indicate that siSLAMF6 alone can modulate CD4^+^ T cell differentiation, but its effect is further amplified when loaded with ERC-Exos (Supplementary Fig. 1).

Next, we examined the involvement of SLAMF6 in more detail. The top ten KEGG-enriched pathways enriched with SLAMF6 are shown in Fig. 4D. SLAMF6 was notably involved in the development of Th17 cells. RORγT is essential for Th17 cell development. In disease models of systemic lupus erythematosus, SLAMF6 promotes the differentiation of T cells into Th17 cells and promotes the recruitment of RORγT to the IL-17A promoter. This action in turn increases the secretion of IL-17A [32]. As a result, we needed to determine whether ERC-siSLAMF6 Exos regulate IL-17A transcription in CD4^+^ T cells. We found that the mRNA expression of RORγT decreased dramatically after treatment with ERC-siSLAMF6 Exos (Fig. 4E). The JASPAR website (https://jaspar.genereg.net/) was used to predict the binding sites for RORγT and IL-17A, and the TTAAATAGGATA sequence was chosen on the basis of the score and strand (Supplementary Table 2). ERC-siSLAMF6 Exos impacted RORγT binding to the IL-17A promoter region, as demonstrated by a chromatin immunoprecipitation assay (Fig. 4F). RORγT is a critical transcription factor involved in the differentiation of Th17 cells [33]. In luciferase reporter assays, RORγT significantly increased the transcriptional activity of IL-17A. However, the introduction of ERC-siSLAMF6 exosomes (Exos) markedly suppressed this RORγT-mediated activation. To confirm the integrity of the luciferase system, IL-22 was used as a positive control. We applied the JASPAR database to predict the binding sites for RORγT and IL-22, leading to the identification of the sequence AAATCTAGGTCA, which was selected for further analysis (Supplementary Table 3). The results demonstrated that ERC-siSLAMF6 Exos effectively inhibited the activation of the IL-17A promoter by RORγT (Fig. 4G). These findings indicate that ERC-siSLAMF6 Exos can stimulate T cell differentiation into Tregs while blocking Th1 and Th17 production. Additionally, we further verified that RORγT binding to the IL17A promoter region was mediated by ERC-siSLAMF6 Exos, which prevented T cells from differentiating into Th17 cells.

### ERC-Exo loaded with siSLAMF6 regulated T cell activation and secretion of inflammatory factors through desialylation

**Fig. 5:**
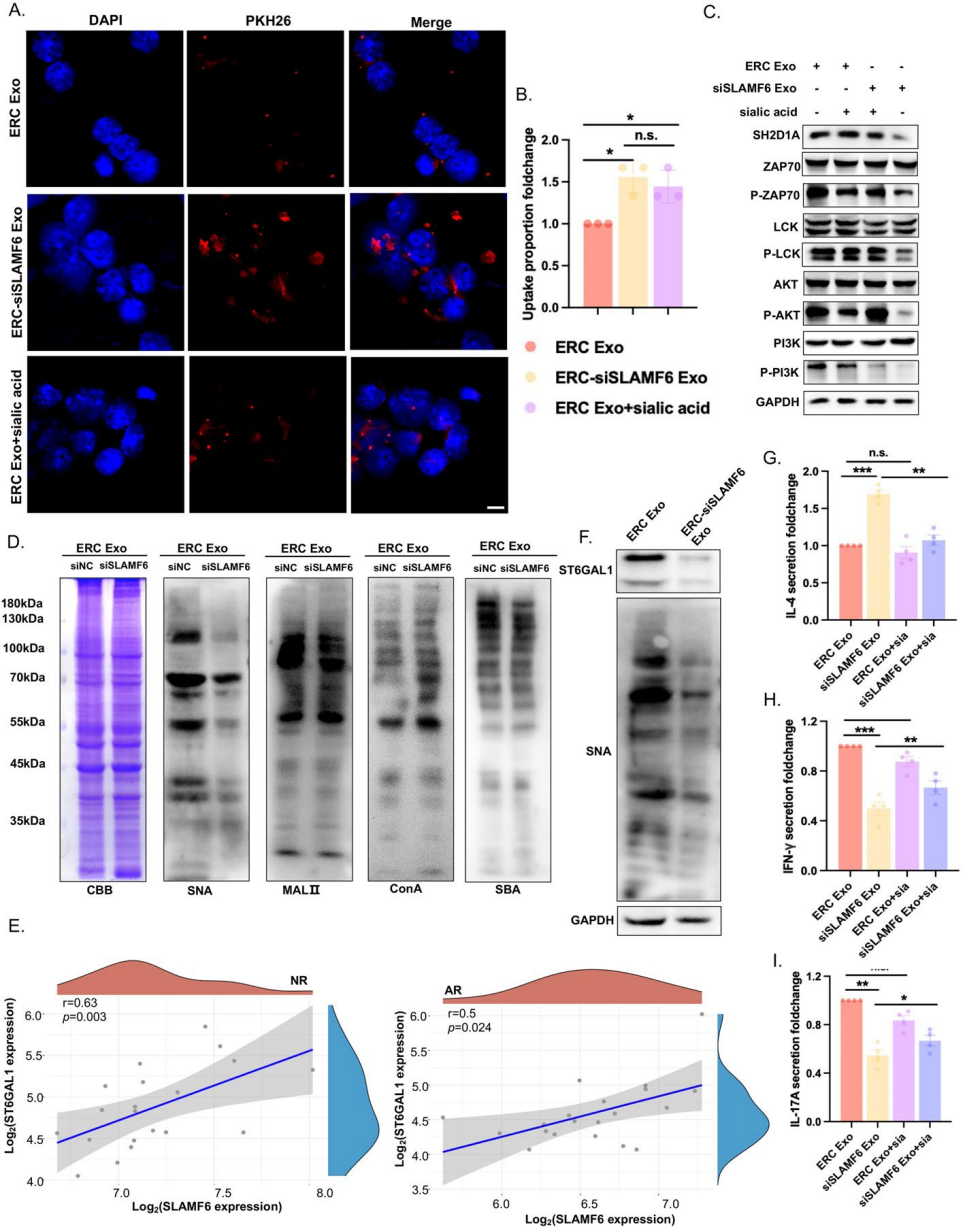
ERC-Exo loaded with siSLAMF6 regulated T cell activation and secretion of inflammatory factors through desialylation. **A** Confocal microscopy was used to detect the uptake of PKH26-labeled Exos by T cells after they were incubated with T cells. Scale bars = 100 μm. **B** Changes in the proportion of exosomes taken up by 20 cells shown in** A** (ERC-siSLAMF6 Exo group versus ERC-Exo group, *P* < 0.05; ERC-siSLAMF6 Exo group versus ERC-Exo + sialic acid group, *P* > 0.05; ERC-Exo group versus ERC-Exo + sialic acid group, *P* < 0.05). **C** Western blot analysis was used to detect the expression of SH2D1A, ZAP70, P-ZAP70, LCK, P-LCK, PI3K, P-PI3K, AKT, and P-AKT in the TCR pathway in CD4^+^T cells cocultured with ERC-Exo or ERC-siSLAMF6 Exo for 48 h. Sialic acid was added for 3 h before the cell protein was harvested to neutralize the sialic acid receptor. GAPDH was used as the loading control. **D** Lectin blot analysis was used to determine the glycosylation levels of SNA, MALII, ConA, and SBA in T cells cocultured with ERC-Exos and ERC-siSLAMF6 Exos. Coomassie brilliant blue (CBB) was used as the loading control. **E** The correlation of SLAMF6 and ST6GAL1 expression was analyzed with Spearman correlation in the acute and no-rejection groups. **F** Western blot analysis was used to detect the protein expression of ST6GAL1 and SNA in T cells cocultured with ERC-Exos or ERC-siSLAMF6 Exos. GAPDH was used as the loading control. **G**–**I** ELISA was used to evaluate the levels of IL-4, IFNγ, and IL-17A secreted by the cell culture supernatant after 48 h of coculture with different groups of exosomes. Sialic acid was added for 3 h to neutralize sialic acid receptors before extraction of the cellular mRNA. n.s., *P* > 0.05, *P < 0.05, **P < 0.01, and ***P < 0.001

Sialylation is a crucial form of glycosylation and influences the occurrence of immunological stress and intestinal inflammation [34, 35]. The sialylated sugar chain structure is a ligand recognized by several types of selectins, and the sugar chain structure is intimately related to exosome recognition and endocytosis [36]. We applied sialic acid to the recipient cells for 3 h to neutralize the sialic acid receptors on the cell surface to explore the mechanism of action of ERC-siSLAMF6 Exos in transplant rejection. We used confocal microscopy to observe the uptake of PKH26-labeled exosomes by CD4^+^ T cells after they were incubated. Exosome uptake by CD4^+^ T cells increased following the use of ERC-siSLAMF6 Exos. Moreover, we blocked the sialic acid receptors on the surface of the recipient cells in advance by exogenously adding sialic acid and found that the uptake of ERC-Exos by CD4^+^ T cells increased, which was consistent with the effect of using ERC-siSLAMF6 Exos alone (Fig. 5A, B). These data indicate that ERC-siSLAMF6 Exos may also promote desialylation in CD4^+^ T cells. Additionally, since exogenous sialic acid neutralizes sialic acid receptors on both the cell and exosome surfaces [36], the use of ERC-siSLAMF6 Exo to induce desialylation is likely a safer and more targeted method. SLAMF6 requires binding to its intracellular adaptor SH2D1A in CD4^+^ T cells to activate the TCR pathway. As shown in Fig. 2D, ERC-Exos highly expressed SLAMF6. Upon the treatment of CD4^+^ T cells with ERC-Exos, the TCR pathway is activated, and this activation is not affected by pretreatment with sialic acid. In contrast, ERC-siSLAMF6 Exos loaded with siSLAMF6 are endocytosed by CD4^+^ T cells, resulting in the inhibition of TCR pathway activation. This finding demonstrated that CD4^+^ T cells are capable of being endocytosed with exosomes and that siSLAMF6 plays a significant role in the function of ERC-siSLAMF6 exosomes (Fig. 5C).

To identify the desialylation site, we performed a lectin blot. ST6β-galactoside α-2,6 sialyltransferase 1 (ST6GAL1) products are α-2,6-sialylated N glycans, and *Sambucus nigra* lectin (SNA) can recognize and bind α-2,6 glycans. The expression of SNA decreased after CD4^+^ T cells were treated with ERC-siSLAMF6 Exos. Moreover, the expression of *Maackia amurensis* lectin II (MALII), concanavalin A (ConA), which is specific for α-Man/α-Glc, and soybean agglutinin (SBA), which preferentially binds to oligosaccharide structures with terminal α- or β-linked *N*-acetylgalactosamine did not significantly differ between the two groups (Fig. 5D). Therefore, ERC-siSLAMF6 Exo desialylates the surface of CD4^+^ T cells by reducing the amount of sialic acid linked to α-2,6 at the end of the N-glycan chain on the cell surface.

ST6GAL1 is a membrane-bound enzyme that mediates α-2,6-linked sialic acid and transfers it to the Galβ1,4-GlcNAc terminus on the cell membrane surface, where it plays a key role in immune responses. Next, we investigated the correlation between SLAMF6 and ST6GAL1 and found that their expression was positively associated with the acute rejection and nonrejection groups. The density curve on the right represents the trend in the distribution of ST6GAL1 expression, and the upper-density curve represents the trend in the distribution of SLAMF6 expression (Fig. 5E). We observed that ERC-siSLAMF6 Exo can also reduce ST6GAL1 protein expression in CD4^+^ T cells, indicating that ERC-Exo loaded with siSLAMF6 inhibits α-2,6-sialylation by influencing the expression of the sialyltransferase ST6GAL1 (Fig. 5F).

Compared with that in the ERC-Exo group, the secretion of the anti-inflammatory factor IL-4 was significantly greater in the ERC-siSLAMF6 Exo group. However, sialic acid reversed this effect (Fig. 5G), while the secretion of the proinflammatory factors IFNγ (Fig. 5H) and IL-17A (Fig. 5I) was significantly reduced. Both of these effects can be reversed by sialic acid. These results reveal that ERC-Exos loaded with siSLAMF6 decrease the amount of sialic acid attached to α-2,6 at the end of the N-glycan chain on the cell surface and increase the number of therapeutic exosomes that endocytose CD4^+^ T cells, further inhibiting T cell receptor signaling pathway activation and thereby affecting immune regulation.

### ERC-Exo loaded with siSLAMF6 significantly attenuated acute cardiac allograft rejection

**Fig. 6:**
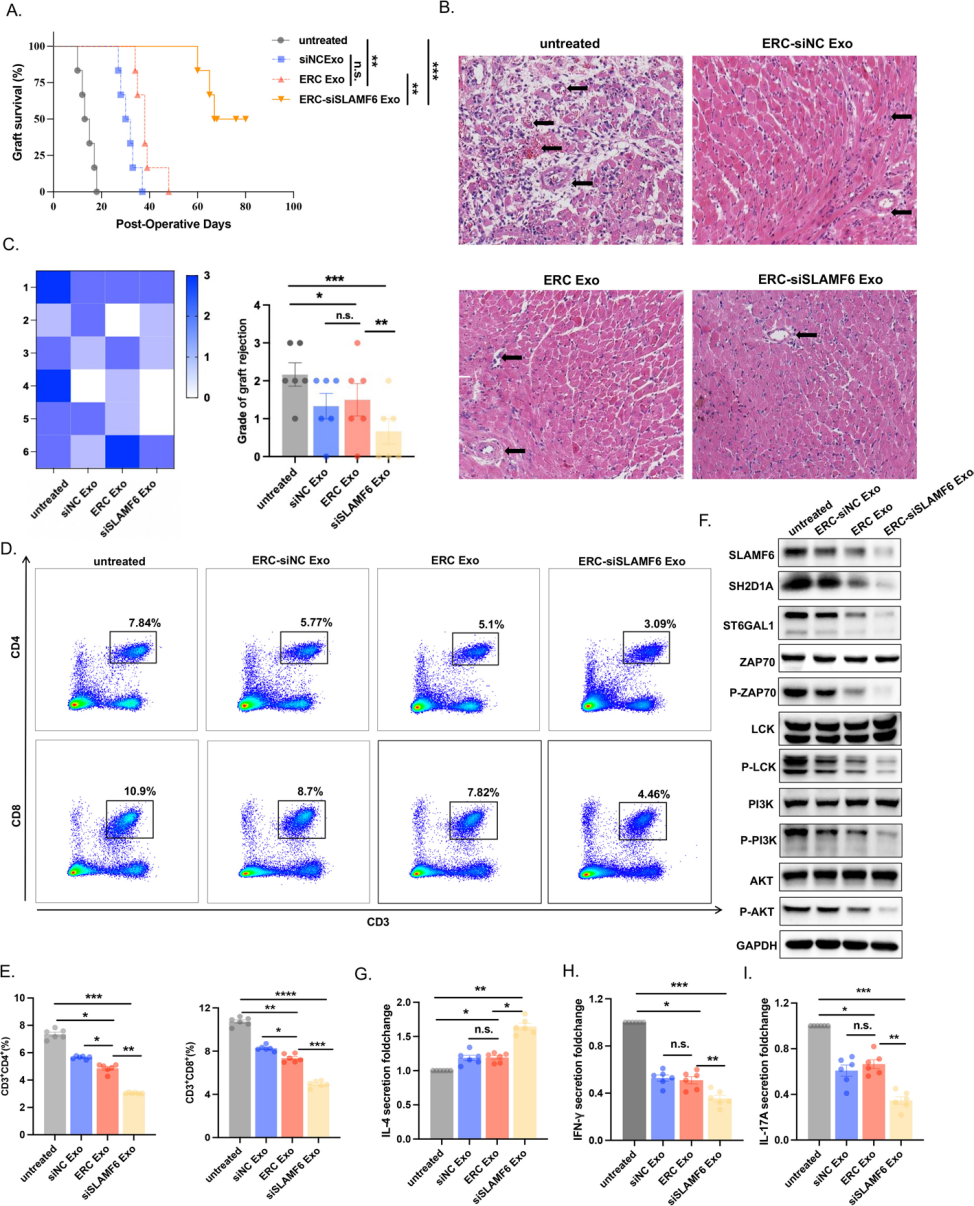
ERC-Exo loaded with siSLAMF6 significantly attenuated acute cardiac allograft rejection through desialylation. **A** The percentage variation in graft survival over time in each C57BL/6 mouse group (*n* = 6). The log‐rank (Mantel–Cox) test was used for statistical analysis (ERC-siSLAMF6 Exo group versus untreated group, *P* < 0.001; ERC0-Exo group versus ERC-siNC Exo group, *P* > 0.05; ERC-siSLAMF6 Exo group versus ERC-Exo group, *P* < 0.01; and ERC-Exo group versus vector group, *P* < 0.01). **B** Representative images of H&E staining of cardiac allograft rejection. The arrows show the perivascular infiltrate with or without necrosis. Scale bars = 100 μm. **C** The rejection score of each group is shown on the left, while the statistical results of the score are shown on the right (ERC-siSLAMF6 Exo group versus untreated group, *P* < 0.001; ERC-Exo group versus ERC-siNC Exo group, *P* > 0.05; ERC-siSLAMF6 Exo group versus ERC-Exo group, *P* < 0.01; and ERC-Exo group versus untreated group, *P* < 0.05). **D** Flow cytometry was used to detect the proportion of CD3^+^CD4^+^ T cells among the recipient splenocytes. The statistical analysis results are shown on the right (*P < 0.05, **P < 0.01, and ***P < 0.001). **E** Flow cytometry was used to detect the proportion of CD3^+^CD8^+^ T cells among the recipient splenocytes. **E** Percentages of CD3^+^CD4^+^ T cells and CD3^+^CD8^+^ T cells; *P < 0.05, **P < 0.01, ***P < 0.001, and ****P < 0.0001. **F** Western blot analysis was used to detect the expression of SH2D1A, ZAP70, P-ZAP70, LCK, P-LCK, PI3K, P-PI3K, AKT, and P-AKT in the TCR pathway in recipient grafts from each group. **G**–**I** ELISA was used to evaluate the levels of IL-4, IFNγ, and IL-17A secreted by recipient serum in each group. n.s., *P* > 0.05, *P < 0.05, **P < 0.01, and ***P < 0.001

In vitro experiments have demonstrated that exosomes generated from ERCs are essential for CD4^+^ T cell differentiation and proliferation. We hypothesized that ERC-siSLAMF6 Exos may enhance the therapeutic effects of ERC-Exos in vivo. We used C57BL/6 recipient mice transplanted with heterotopic hearts from BALB/c donor mice to determine whether ERC-siSLAMF6 Exos play a similar immunoregulatory role in vivo. Exosomes were injected into the recipient mouse tail vein. We noted that siSLAMF6-loaded ERC-Exos significantly prolonged allograft survival (Fig. 6A). The heart allografts were collected 8 days after transplantation and evaluated by H&E staining. Vasculitis and extensive infiltration of inflammatory cells were among the significant rejections observed in the untreated group. Graft rejection was mitigated by the use of ERC-siNC Exos and ERC-Exos, while ERC-siSLAMF6 further reduced rejection (Fig. 6B). The results of the microscopic examination were validated by H&E staining scores, in the untreated group, all six mice exhibit varying degrees of transplant rejection. One mouse in both the ERC-siNC Exo and ERC-Exo groups, and two in the ERC-siSLAMF6 Exo group, did not experience acute transplant rejection. Additionally, treatment with ERC-siSLAMF6 Exo was observed to reduce the severity of transplant rejection (Fig. 6C).

T cell proliferation in the early stages of transplant rejection can indicate the severity of acute cellular rejection. As a result, we extracted spleen cells from recipient mice for flow cytometry analysis and discovered that the proportions of CD3^+^CD4^+^ T cells and CD3^+^CD8^+^ T cells in the untreated group were considerably greater than those in the ERC-siNC Exo and ERC-Exo treatment groups, whereas the proportions of CD3^+^CD4^+^ T cells and CD3^+^CD8^+^ T cells in the ERC-siSLAMF6 Exo group were the lowest (Fig. 6D, E).

To determine whether ERC-siSLAMF6 Exos modify heart allografts, we validated the protein expression of the collected allograft-derived proteins. The expression of the sialyltransferase ST6GAL1, which is critical for the process of desialylation, was significantly decreased in the ERC-siSLAMF6 Exo treatment group, and TCR pathway activation was also significantly suppressed (Fig. 6F). After that, we collected serum from the recipient mice to examine changes in proinflammatory and anti-inflammatory cytokine production levels. The secretion level of the anti-inflammatory factor IL-4 was the lowest in the untreated group and the highest following treatment with ERC-siSLAMF6 Exo (Fig. 6G). The levels of the proinflammatory factors IFNγ (Fig. 6H) and IL-17A (Fig. 6I) were highest in the untreated group and lowest following treatment with ERC-siSLAMF6 Exos. These findings suggest that ERC-derived exosomes can minimize graft rejection in vivo and that ERC-siSLAMF6 Exos can even improve this effect.

### ERC-Exo loaded with siSLAMF6 regulated acute transplant rejection-related T cell differentiation in vivo

**Fig. 7:**
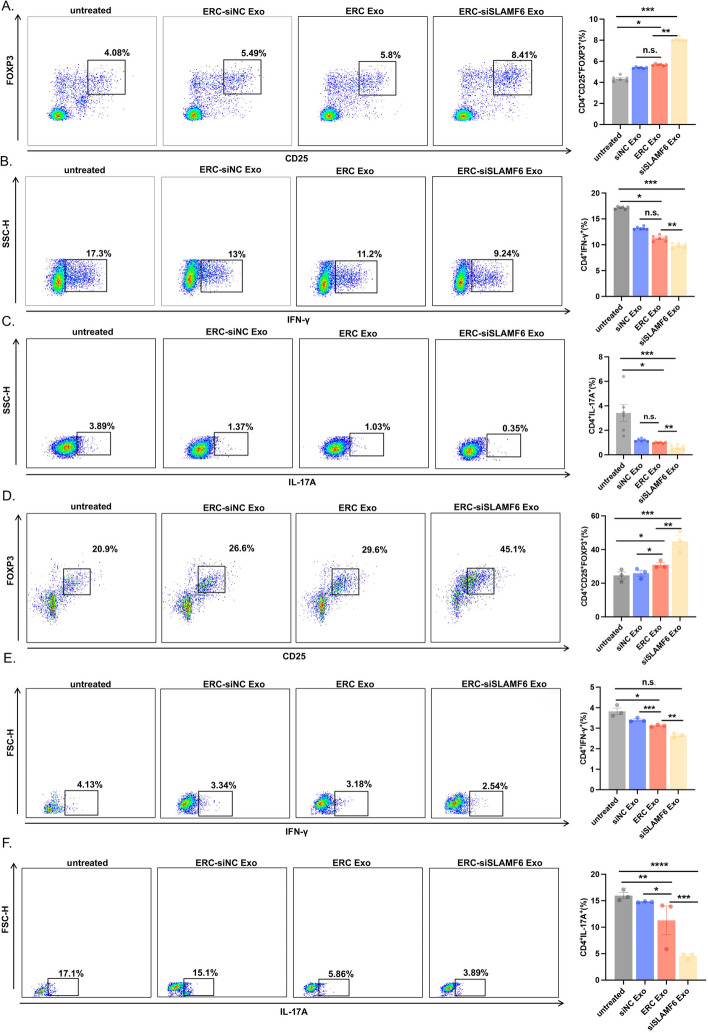
ERC-Exo loaded with siSLAMF6 regulated acute transplant rejection-related T cell differentiation in vivo*.* **A** Flow cytometry was used to detect the proportion of Tregs in recipient splenocytes. The statistical analysis results are shown on the right (ERC-siSLAMF6 Exo group versus untreated group, *P* < 0.001; ERC-Exo group versus ERC-siNC Exo group, *P* > 0.05; ERC-siSLAMF6 Exo group versus ERC-Exo group, *P* < 0.01; and ERC-Exo group versus untreated group, *P* < 0.05). **B** Flow cytometry was used to detect the proportion of Th1 cells in recipient splenocytes. The statistical analysis results are shown on the right (ERC-siSLAMF6 Exo group versus untreated group, *P* < 0.001; ERC-Exo group versus ERC-siNC Exo group, *P* > 0.05; ERC-siSLAMF6 Exo group versus ERC-Exo group, *P* < 0.01; and ERC-Exo group versus untreated group, *P* < 0.05). **C** Flow cytometry was used to detect the proportion of Th17 cells in the recipient splenocyte population. The statistical analysis results are shown on the right (ERC-siSLAMF6 Exo group versus untreated group, *P* < 0.001; ERC-Exo group versus ERC-siNC Exo group, *P* > 0.05; ERC-siSLAMF6 Exo group versus ERC-Exo group, *P* < 0.01; and ERC-Exo group versus untreated group, *P* < 0.05). **D** Flow cytometry was used to detect the proportion of Tregs in heart mononuclear cells. The statistical analysis results are shown on the right (ERC-siSLAMF6 Exo group versus untreated group, *P* < 0.001; ERC-Exo group versus ERC-siNC Exo group, *P* < 0.05; ERC-siSLAMF6 Exo group versus ERC-Exo group, *P* < 0.01; and ERC-Exo group versus untreated group, *P* < 0.05). **E** Flow cytometry was used to detect the proportion of Th1 cells in heart mononuclear cells. The statistical analysis results are shown on the right (ERC-siSLAMF6 Exo group versus untreated group, *P* < 0.001; ERC-Exo group versus ERC-siNC Exo group, *P* > 0.05; ERC-siSLAMF6 Exo group versus ERC-Exo group, *P* < 0.01; and ERC-Exo group versus untreated group, *P* < 0.05). **F** Flow cytometry was used to detect the proportion of Th17 cells among heart mononuclear cells. The statistical analysis results are shown on the right (ERC-siSLAMF6 Exo group versus untreated group, *P* < 0.0001; ERC-Exo group versus ERC-siNC Exo group, *P* < 0.05; ERC-siSLAMF6 Exo group versus ERC-Exo group, *P* < 0.001; and ERC-Exo group versus untreated group, *P* < 0.01)

Tregs, Th1 cells, and Th17 cells play essential roles in transplant rejection. As a result, we performed flow cytometric analysis on splenocytes and discovered that the percentage of Tregs was decreased in the untreated group and increased following ERC-siNC Exo and ERC-Exo therapy. The proportion of Tregs increased significantly after treatment with ERC-siSLAMF6 Exos (Fig. 7A). Next, we wanted to determine whether treatment with ERC-siSLAMF6 Exos would reduce transplant rejection by influencing T cell differentiation. We counted and stained the splenocytes of the recipient mice and discovered that the proportion of Th1 cells was highest in the untreated group. The proportion of Th1 cells decreased when ERC-siNC Exos or ERC-Exos were injected. The proportion of Th1 cells decreased considerably following ERC-siSLAMF6 Exo treatment (Fig. 7B). Moreover, we detected the same phenomenon in Th17 cells, and the function of ERC-siSLAMF6 Exos was confirmed in vitro (Fig. 7C).

To determine whether ERC-siSLAMF6 Exos affect the heart, we applied Percoll density gradient centrifugation to separate cardiac mononuclear cells from transplant recipient mice. We discovered similar results in splenocytes by labeling and flow cytometry, indicating that the use of ERC-siSLAMF6 Exos can increase the number of Tregs (Fig. 7D) while decreasing the number of Th1 (Fig. 7E) and Th17 cells (Fig. 7F). These results suggest that exosome treatment has a positive effect on target organs and that applying ERC-siSLAMF6 Exos significantly improves the therapeutic efficacy of ERC-Exos and further attenuates transplant rejection.

## Discussion

Approximately 10–12% of organ transplant recipients experience acute rejection even with immunosuppressive treatment [37]. As a result, better treatments with fewer side effects are needed. Owing to their anti-inflammatory and immunomodulatory properties, stem cells and their resulting extracellular vesicles (EVs) have emerged as a new area of study. ERCs are mesenchymal-like stem cells. They have the advantages of being noninvasive and having a high rate of proliferation. Several studies have confirmed that the therapeutic activity of ERC is mediated by extracellular vesicles [38]. There have been no investigations on the use of ERC-derived exosomes for the treatment of transplant rejection. To date, enhancing the therapeutic efficacy of stem cell-derived exosomes has been a central focus of exosome research, with the integration of gene therapy emerging as a leading strategy. In conventional gene therapy, the natural uptake of nucleic acids by cells is inefficient, and these nucleic acids are prone to degradation and clearance. To overcome the limitations of nucleic acid carriers, various nonviral methods have been developed, including calcium phosphate coprecipitation, cationic lipids, gene guns, electroporation, and microinjection [39]. Despite their potential, these technologies remain largely confined to preclinical studies and have yet to demonstrate clinical translatability. Exosomes exhibit low immunogenicity and actively participate in the intercellular exchange of DNA, RNA, proteins, and other cellular components. They are also capable of transmitting genetic information across the blood–brain barrier.

Small interfering RNA (siRNA) is a widely used gene-silencing tool with the ability to inhibit specific target genes selectively, demonstrating significant potential in gene therapy [40]. However, owing to their susceptibility to degradation by RNases in the body, the development of a safe and efficient siRNA delivery system is crucial. Exosomes are considered optimal vehicles for gene therapy because of their natural, nonsynthetic, and nonviral components. Their small size enables them to traverse major biological membranes, while their bilipid structure provides a protective environment for the RNA and proteins they carry, preventing degradation and thereby facilitating efficient delivery to recipient cells [41]. Importantly, the miRNAs carried by exosomes are known to participate in multiple signaling pathways [42]. Consequently, determining whether the artificial engineering of cells to produce exosomes influences the expression of endogenous miRNAs within exosomes is essential. In a study investigating the treatment of osteoporosis using exosome-delivered siShn3, the authors employed next-generation sequencing and confirmed that there was no significant difference in miRNA expression between the Exo-siShn3 and Exo groups. This finding suggests that artificial modification does not affect the endogenous miRNA content of exosomes [43]. These results provide a basis for our subsequent research, in which we will sequence the miRNA expression profiles of ERC-Exo and ERC-siSLAMF6. We aimed to further investigate whether miRNA-enriched pathways play a role in the occurrence of acute transplant rejection. In this study, we found that ERC-derived exosomes can reduce the occurrence of allogeneic rejection by reducing CD4^+^ T cell proliferation and the development of CD4^+^ T cells into Th1 and Th17 cells while increasing CD4^+^ T cell differentiation into Tregs. This phenomenon is associated with our observations in other disease models [44].

The signaling lymphocyte activation molecule (SLAM) family comprises transmembrane coreceptors that play a critical role in modulating antigen-driven T cell responses. Signaling pathways downstream of SLAM receptors are mediated primarily by the adaptor protein known as SLAM-associated protein (SH2D1A). Proper regulation of SLAM-SH2D1A signaling in T cells is essential for maintaining immune homeostasis. Dysregulation, whether due to insufficient or excessive signaling, can lead to the development of autoimmune diseases. SLAMF6 is extensively expressed in immune cells and plays an immunomodulatory role in the cross-talk among several immune cells. The extracellular domain of SLAMF6 functions as its own autoligand, and the T cell signaling mediated by this binding is regulated by SH2D1A. SH2D1A is expressed predominantly in T cells and natural killer cells and binds to the immunoreceptor tyrosine-based switch motif (ITSM) in the cytoplasmic tail of SLAMF6, thereby activating the T cell signaling pathway. When SLAMF6 binds to SH2D1A, it recruits lymphocyte-specific protein tyrosine kinase (LCK), thereby promoting the activation of the T cell receptor signaling pathway [45]. Additionally, this interaction facilitates the nuclear translocation of RORγT, leading to the transcriptional activation of IL-17A [46]. One of the characteristics of the adaptive immune response is the differentiation of CD4^+^ T helper (Th) cells into different effector cell types. Studies have confirmed that SLAMF6 stimulates Th cells to differentiate into a Th1 phenotype [47]. We used bioinformatics to explore clinical data related to nonacute and acute transplant rejection from the GEO public database, and we discovered that SLAMF6 is overexpressed in acute transplant rejection tissues. During acute transplant rejection, KEGG pathway analysis revealed that the Th1, Th2, and Th17 cell differentiation pathways and the T cell receptor (TCR) signaling pathway are activated. SLAMF6 clustering has been found to stimulate T cells and is needed for TCR activation [48]. SLAMF6 has also been shown to have distinct clinical uses in X-linked lymphoproliferative diseases, systemic lupus erythematosus, and melanoma [49–51]. The majority of these clinical values are obtained by influencing B and T cells [52]. These bioinformatics results verified that SLAMF6 can promote the occurrence of allograft rejection.

Th1 and Th17 immune responses are involved in the development of acute allograft rejection [53], and Tregs play important roles in moderating immunological responses and the proinflammatory microenvironment [54]. The dysregulation of the Th17/Treg balance has been associated with the onset and progression of a variety of diseases, including autoimmune diseases, inflammatory diseases, and malignancies [55]. As a result, exploring the interactions between SLAMF6 and Th1, Th17, and Tregs in transplant rejection is critical. To obtain exosomes containing siSLAMF6, ERCs were isolated and transfected with siSLAMF6. Experiments in vivo and in vitro demonstrated that ERC-Exos loaded with siSLAMF6 can reduce the occurrence of allogeneic rejection. In addition, ERC-siSLAMF6 Exo treatment can enhance the therapeutic impact of ERC-Exos. Our study demonstrated that siSLAMF6 alone can influence CD4^+^ T cell differentiation, although its efficacy is not as pronounced as that of ERC-siSLAMF6 Exos. However, it remains unclear whether transfection with siSLAMF6 alters the protein and nucleic acid contents of ERC-Exos themselves. This influence will be a subject of future investigations.

The most common posttranslational modification involved in immunological responses is glycosylation [56]. Sialylation is a type of glycosylation in which sialic acid is attached to glycans via α-2,3-, α-2,6-, and α-2,8-linked ends [35]. For immune evasion, hypersialylation relies on protein–glycan interactions [57]. Hypersialylation can increase T cell proliferation and disease severity in ulcerative colitis [58]. In this study, we first neutralize sialic acid receptors on the surface of receptor cells by exogenously adding sialic acid. The uptake of exosomes by recipient cells increased, which was the same as that observed in cells treated with ERC-siSLAMF6 Exos alone. These findings indicate that ERC-siSLAMF6 Exos play a role in desialylation modification on the surface of receptor cells. Using lectin blot analysis, we found that only the expression of SNA was suppressed in cells treated with ERC-siSLAMF6 Exos. Clusterin SNA detects and binds α-2,6-linked sialic acid at the N-sugar chain terminus. ST6GAL1 catalyzes the transfer of α-2,6-sialic acid to the Gal1,4-GlcNAc terminus on the cell surface and the secretion of glycans [59]. It has been reported that neutralizing sialic acid receptors on the surface of dendritic cells with sialic acid can increase their phagocytic ability [60]. Similarly, after the desialylation of microglia, their phagocytosis of neurons is significantly increased [61]. On the basis of these findings, we hypothesize that the specific recognition and endocytosis of exosomes by receptor cells may be influenced by the sialylated glycan structures present on the surface of these cells. We also acknowledge that treating with sialic acid may directly induce phenotypic changes in receptor cells, a possibility that we will investigate in future studies.

ST6GAL1 inhibition increases lung inflammation, promotes arthritis development, and suppresses ulcerative colitis development [58, 62, 63]. This study revealed that after recipient cells were treated with ERC-siSLAMF6 Exos, the mRNA and protein expression of ST6GAL1 was reduced. ERC-Exo loaded with siSLAMF6 reduces the sialic acid connected to α-2,6 at the end of the N-glycan chain on the CD4^+^ T cell surface, increases the number of therapeutic exosomes endocytosed into CD4^+^ T cells, inhibits the activation of T-cell receptor signaling pathways, decreases the secretion of proinflammatory factors, and increases the secretion of anti-inflammatory factors.

**Fig. 8:**
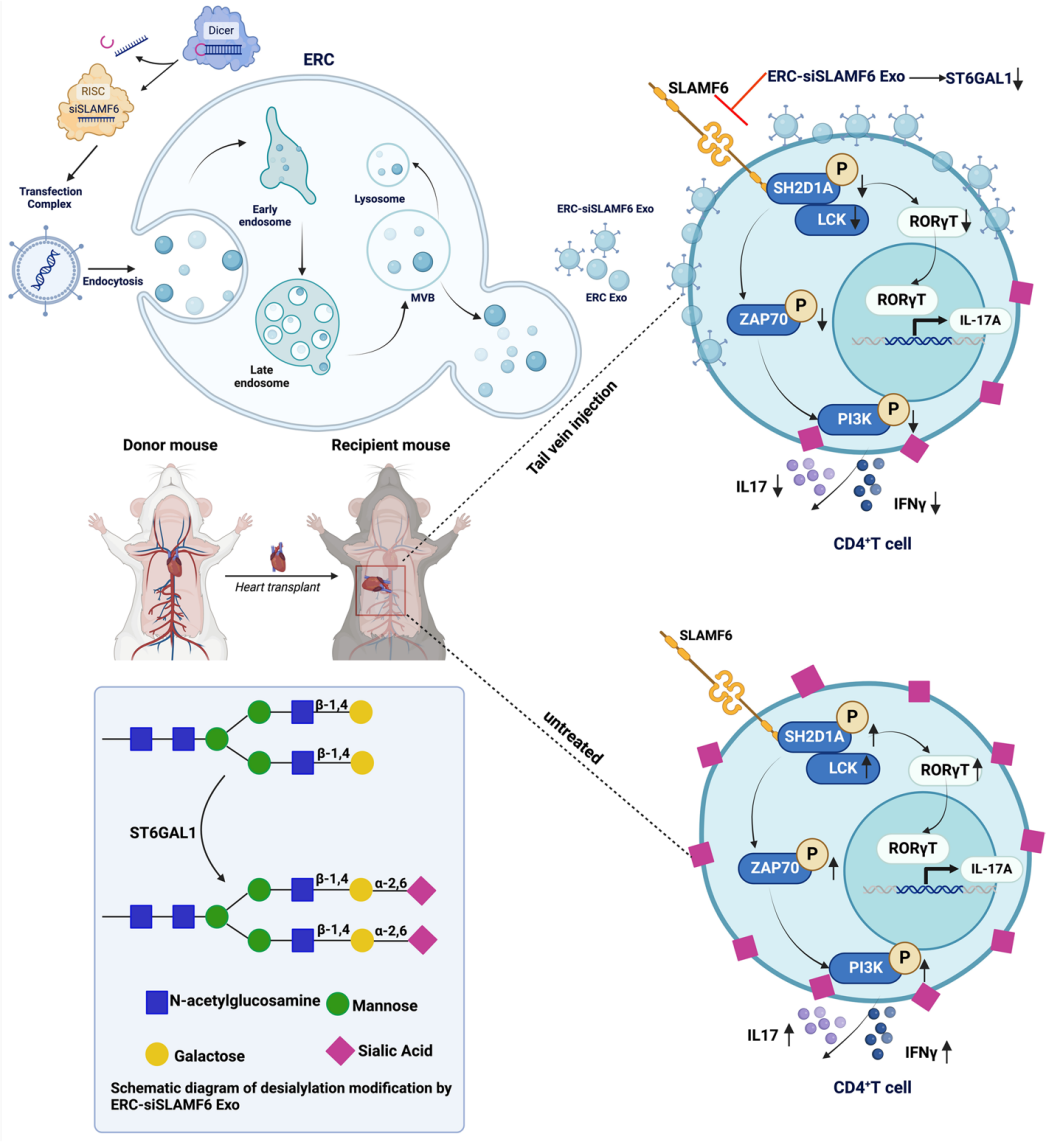
Schematic diagram of this study. ERC-siSLAMF6 Exos play a role in desialylation. In brief, ERC-siSLAMF6 Exos can reduce sialic acid residues at the termini of N-linked glycan chains on the cell surface, which are typically connected by α-2,6 bonds. This reduction facilitates increased uptake of therapeutic exosomes by CD4^+^ T cells, thereby preventing the activation of the TCR signaling pathway and inhibiting the binding of RORγT to the IL-17A promoter region. As a result, CD4^+^ T-cell proliferation and differentiation into Th1 and Th17 subsets are suppressed, while the generation and proliferation of Tregs are promoted. These effects collectively contribute to the effective inhibition of acute transplant rejection. This schematic diagram was created with BioRender.com

In this study, we demonstrated that ERC-derived exosomes can act as natural carriers for loaded siSLAMF6. ERC-siSLAMF6 Exo treatment can improve the therapeutic efficacy of ERC-Exo for acute allograft rejection, suggesting a novel therapeutic target. Building on previously reported mechanisms, we confirmed for the first time that ERC-siSLAMF6 Exos reduce CD4^+^ T cell activation by downregulating the production of the sialyltransferase ST6GAL1. This reduction leads to decreased levels of α-2,6-linked sialic acid on the N-glycan termini of the cell surface, resulting in desialylation. Consequently, the uptake of exosomes by CD4^+^ T cells is increased, which inhibits the activation of the T cell receptor signaling pathway and prevents RORγT from binding to the IL-17A promoter region. This chain of events suppresses the proliferation of CD4^+^ T cells, inhibits their differentiation into Th1 and Th17 cells, and promotes their differentiation into Tregs (Fig. 8).

## Supplementary Information

Supplementary Material 1.

Supplementary Material 2.

Supplementary Material 3.

Supplementary Material 4.

## Data Availability

The datasets referenced to support the conclusions of this research are included in the article and its supplementary files.

## Abbreviations

SLAMF6: Signaling lymphocyte activation molecule family 6
MSCs: Mesenchymal stromal cells
ERCs: Endometrial regenerative cells
EVs: Extracellular vesicles
siRNA: Small interfering RNA
Th cells: T helper cells
Tregs: Regulatory T cells
Co-IP: Coimmunoprecipitation
HE: Hematoxylin–eosin
GEO: Gene Expression Omnibus
KEGG: Kyoto Encyclopedia of Genes and Genomes
TEM: Transmission electron microscope
NTA: Nanoparticle tracking analysis
TCR: T cell receptor
IFN-γ: Interferon-γ
ST6GAL1: ST6 β-galactoside α-2,6 sialyltransferase 1
SNA: *Sambucus nigra* Lectin
MALII: *Maackia amurensis* Lectin II
ConA: Concanavalin A
SBA: Soybean agglutinin
