# Supplementary figures and images for: Endometrial regeneration cell-derived exosomes loaded with siSLAMF6 inhibit cardiac allograft rejection through the suppression of desialylation modification
Source: Cell Mol Biol Lett. 2024 Oct 1;29:128. doi: 10.1186/s11658-024-00645-y (PMC11443917; doi:10.1186/s11658-024-00645-y)

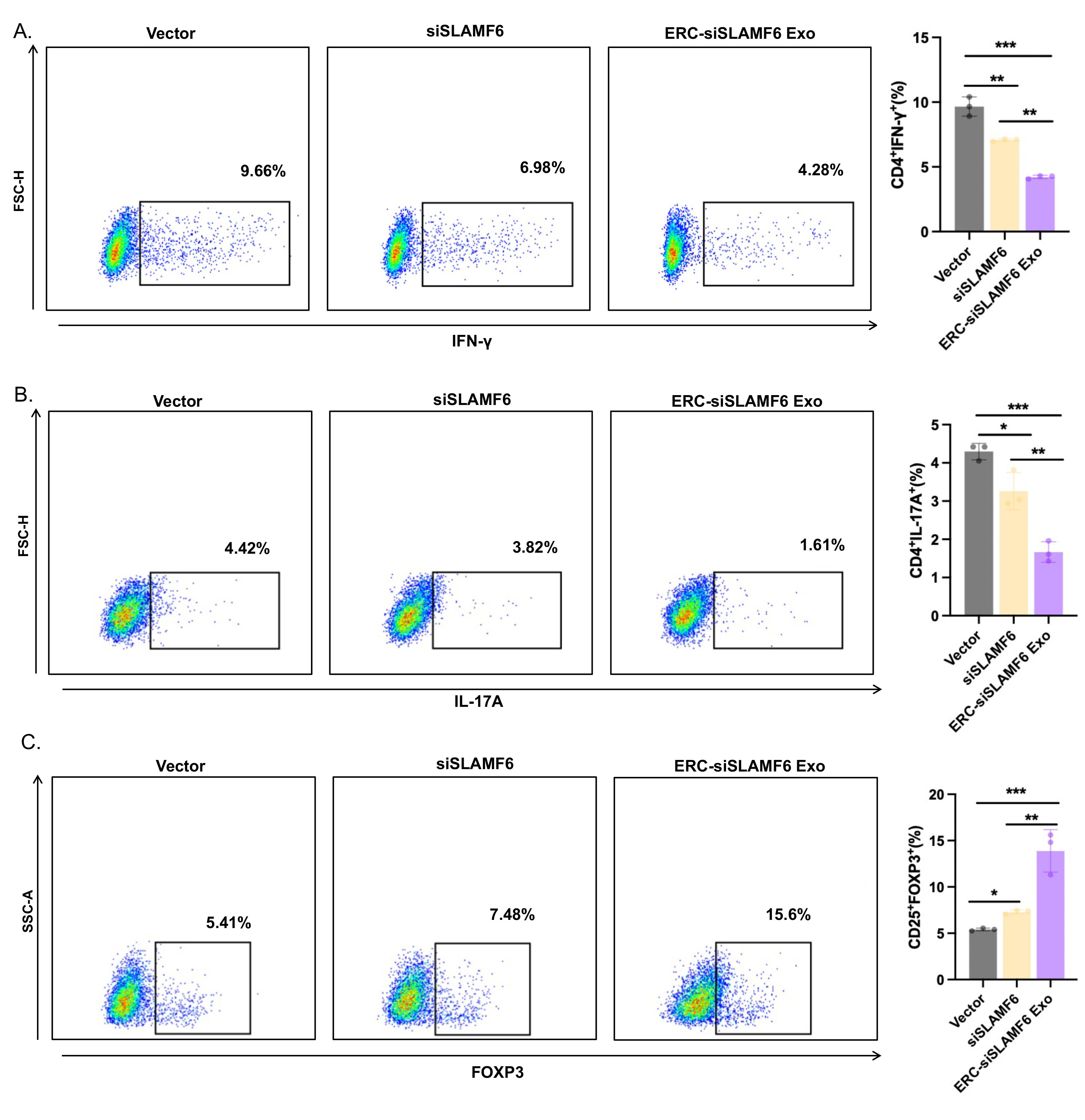

Supplement: Supplementary file 1 — Supplementary Material 1. [file 11658_2024_645_MOESM1_ESM.jpg]
